# Application of Gutta Percha to Dental Purposes

**Published:** 1851-01

**Authors:** 


### ARTICLE IV.

### *Application of Gutta Percha to Dental Purposes.*

The peculiar properties of this remarkable substance, familiar, doubtless to all the readers of the Journal, have rendered it extremely useful in the arts, for a variety of purposes. It struck me at once, on first finding that it could so readily be softened, and again almost instantaneously rendered hard, that it might be found very useful in our own profession. I made some experiments with it, and now find it, for a great many purpoess, so useful in my practice, as to be quite unwilling to dispense with it.

My first attempts with gutta percha were to use it as a substitute for wax, in taking impressions of the mouth. I took hold of it quite sanguinely for this purpose, but was somewhat disappointed in the results obtained. The fact, that at a certain temperature it becomes quite elastic, renders it necessary that it should be kept in the mouth, in place, till it cools below this point. As this requires some time, even with the aid of ice to hasten the cooling, it renders it less convenient than wax for the purpose. Indeed, since the time I first employed it for taking impressions, some improvements have been introduced, in this city at least, (and which may be described at some future time,) which, for partial cases, renders wax all that can be desired for the purpose. I can easily imagine, too, that if any considerable number of teeth were left in the mouth, and the gutta percha allowed to remain a little too long, it would be found extremely difficult to remove. For partial cases, I have, for these and other reasons, ceased to use it, although, from some communications in the Journal, it seems to have been been used with better success by other members of the profession. But, although I did not find it so valuable as I anticipated for the purpose here indicated, for taking impressions for an entire set, I know of nothing which will answer as a substitute.

My method of using it for this purpose is the following: An impression is taken with wax, in the usual way, and a plaster model made from the impression. From this model a gutta purcha plate, an eighth of an inch in thicknes, is made, precisely in the form intended to be given to the gold plate afterward. This is tried in the mouth, examined closely, and such parts as are not found to fit well are softened, a small portion at a time, and whilst the whole is held firmly with the left hand, the part softened is pressed against the mouth with pounded ice in the end of a napkin, held in the right hand. By going over the plate or frame in this manner, an impression may at last be obtained which will be very accurate. The ice must, of course, be held in contact with the gutta percha till the latter is perfectly hardened.

We have been making cavity plates in this city, differing both from Gilbert's plan and that of Dr. Cleaveland. We have found it to answer well—better, in our hands, than any other cavity plate. The chamber is made by cutting out a piece of the plate formed in the ordinary way, in the form intended to be given to the cavity; and the cavity is made by soldering on an additional piece, as is done in Cleaveland's plate, but without wire, or anything to give additional thickness to the edge of the cavity. I mention this manner of making cavity plates incidentally, at this time, simply to enable me to describe the manner I have fallen upon for taking the impression for making the plate. From a plaster model, made in the usual way, after a wax impression, I get the gutta percha plate above described, and cut out from it a piece in the form intended to be given to the cavity. I then, after fitting the gutta percha plate in every other part, soften by touching it with a napkin dipped in boiling water, to a little distance from the edge of the cavity all round, and hold it in place with the ice, as above directed, till it becomes hard. The advantage of this method is very obvious: it enables you to get, with uniform certainty, a perfectly accurate impression of the part upon which, and upon which alone, the adhesion of the plate depends. It is much better than the lead plate recommended for the same purpose, for the parts which fit well cannot spring in the slightest degree whilst you are perfecting the adaptation of the other parts. Since adopting the method described, I have experienced very little trouble in getting atmospheric plates to adhere as firmly as can be desired.

In taking an impression of the lower jaw, it is simply necessary to cut out a piece of sheet gutta percha, as near the size of the mouth in hand as possible, soften and mould it to the part, trimming away, with scissors, the superabundant portions.

In my unsatisfactory trials to substitute gutta percha for wax, in taking impressions, I found it to answer so admirable a purpose as a wax-holder, that I have never, since then, used any other, when I found one of these articles desirable. Every one who has used the ordinary tin frame, knows how impossible it is to get them to answer well in all the variety of cases which present themselves, no matter how large his assortment may be. The method of using gutta percha for this purpose is exceedingly simple and easy. Take about as much gutta percha in quantity as you would use of wax for taking an impression. Soften and use it precisely as you would use wax without a frame to hold it. As soon as you remove it from the mouth, (which, in this case, may be done as soon as you obtain the impression,) enlarge it a little with your fingers, at the part corresponding to the labial portion of the gum, and stretch it upward; then throw it into cold water for a few minutes, and you have a wax-holder perfectly adapted to the case in hand. A very small quantity of wax in this frame, will enable you to get a good impression. The advantages of gutta percha for this purpose are so very oovious, that I have no doubt it has been employed in the same way by other practitioners. If it were useful for no other purpose, I find it so convenient and advantageous for this, that I would not be without it for twenty times its cost.

In using gutta percha, exclusively, for taking impressions in partial cases, I generally proceeded as follows: After preparing the holder as you would do if wax were used, soften the requisite quantity of gutta percha, by allowing it to remain a few minutes in water kept at the boiling point, and then place it in the holder. Before putting it in the mouth, immerse it for an instant in cold water; this will prevent it from adhering to the teeth and lips, which otherwise it would be apt to do. Put it in the mouth, and press steadily and firmly against the part of which you wish to obtain the impression. Hold it steadily in place for a minute or two, then remove and place in a glass of ice water, which it will be well to have by you on your stand. Allow it to remain only long enough for the surface to be chilled, for which a minute or two will be sufficient. Then return it to its place in the mouth. This you can readily do, if you are cautious, without injury to the impression. In this way you may remove and replace it, till you are satisfied that the contraction is complete. I think it well always to observe this precaution, for, even if the patient could allow it to remain in the mouth sufficiently long for complete contraction to take place, its contraction on the teeth in some cases would render it very difficult to remove, especially where the teeth happened to be larger at the crowns than the necks. The best way that such an accident could be remedied, would be to apply, repeatedly, napkins dipped in boiling water, till the impression is sufficiently softened to be removed. I have said that I had ceased to use gutta percha for this purpose, because I had found wax more convenient; still, I am not satisfied that it might not be used with advantage, and give the result of my experience to those who have not used it at all and may be disposed to try it. After an impression is obtained, it enables you to procure a very beautiful plaster model.

A most excellent cement for arranging teeth on the plate, so that they may be tried in the mouth before soldering, (a practice invariably pursued by me,) may be made of this useful substance. For this purpose, it is combined with resin, in the proportions: three parts resin to one of gutta percha. The resin is first melted with a gentle heat, and the gutta percha dropped into it in small pieces, when it will be found rapidly to dissolve. I have used several other compositions for this purpose, but none with the same degree of satisfaction. It adheres with great tenacity to the plate, when heated and dropped upon it; it is not at all affected by the moisture of the mouth; it can, with a low degree of heat, be so softened as to allow the teeth to be moved with ease; and when this is to be done, can be chipped away from the plate with as much ease as shellac. But it is readily made, and any one who desires an article of this kind, will soon be able to test its value.

This cement will be found valuable for another purpose. Those who practice the operation of destroying the dental pulp with arsenic, know that it is very desirable that the preparation used should be well secured in the decayed cavity, without being allowed to press upon the exposed pulp; for, when this occurs, severe pain will inevitably ensue. To accomplish this object, a number of valuable methods have been proposed; but generally the cement here described will save both time and trouble, and will perfectly answer the desired purpose. After the preparation is applied over the exposed pulp, place in the cavity, loosely, some pieces of the cement, allowing them to project above its edges; touch it with an instrument slightly heated, and it will quickly soften, and attach itself to the sides of the cavity. This will perfectly fill the cavity, and exclude every particle of moisture so well that the cotton used for applying the preparation will be found, on its removal, exactly in the same condition as when it was put in place.

A little more than a year since, I had the misfortune to sprain the second joint of the thumb of my right hand. It is quite unnecessary to say to the readers of this Journal, that few more serious accidents could well happen to a practicing dentist. The sprain was a slight one, but the absolute necessity I was under, at the time, of using my hand almost incessantly, not only prevented it from getting well, but injured the joint so much that I was at last entirely disabled. Every one knows how tedious an affair a sprain is, even when the strained ligaments are allowed absolute rest. I could not afford to lay by a single day, and yet I could not operate at all. In this gloomy extremity, after considerable loss of time, it occurred to me, that if the joint could be supported by any means, which would not interfere with the use of the rest of my hand, I might get on tolerably well at least. I had then used gutta percha, and it occurred to me that this was just what I wanted. I went into my work-shop, moulded a piece of gutta percha to the joint, making a band around the thumb below the joint, and extending the other extremity to a point just below the joint at the wrist. Through this tongue, a hole was made for a piece of ribbon, which was tied round the wrist. The whole was covered with silk, which is easily accomplished by warming the surface of the gutta percha, and sticking the silk to it. I put it on, went to work, and have not since, from this cause, been prevented a single hour from operating. This is an accident to which, of course, every one is liable, and I thought an account of this simple mode of relief in such a case, might prove useful to some person.

I have used the substance which has formed the subject of this article for a great variety of trifling purposes, which are probably scarcely worth describing formally, but which certainly contribute very much to the conveniences of my work-shop. I am sure it will be found very useful in many ways that are scarcely suspected now, if attention is once seriously turned to it. A.

WASHINGTON, D. C.

